# Neutrophil Extracellular Traps Enhance Early Inflammatory Response in Sendai Virus-Induced Asthma Phenotype

**DOI:** 10.3389/fimmu.2016.00325

**Published:** 2016-08-26

**Authors:** Antonina Akk, Luke E. Springer, Christine T. N. Pham

**Affiliations:** ^1^Department of Medicine, Division of Rheumatology, Washington University School of Medicine, Saint Louis, MO, USA

**Keywords:** neutrophils, NETs, cytokines, dipeptidyl peptidase I, Sendai virus-induced asthma

## Abstract

Paramyxoviral infection in childhood has been linked to a significant increased rate of asthma development. In mice, paramyxoviral infection with the mouse parainfluenza virus type I, Sendai virus (Sev), causes a limited bronchiolitis followed by persistent asthma traits. We have previously shown that the absence of cysteine protease dipeptidyl peptidase I (DPPI) dampened the acute lung inflammatory response and the subsequent asthma phenotype induced by Sev. Adoptive transfer of wild-type neutrophils into DPPI-deficient mice restored leukocyte influx, the acute cytokine response, and the subsequent mucous cell metaplasia that accompanied Sev-induced asthma phenotype. However, the exact mechanism by which DPPI-sufficient neutrophils promote asthma development following Sev infection is still unknown. We hypothesize that neutrophils recruited to the alveolar space following Sev infection elaborate neutrophil extracellular traps (NETs) that propagate the inflammatory cascade, culminating in the eventual asthma phenotype. Indeed, we found that Sev infection was associated with NET formation in the lung and release of cell-free DNA complexed to myeloperoxidase in the alveolar space and plasma that peaked on day 2 post infection. Absence of DPPI significantly attenuated Sev-induced NET formation *in vivo* and *in vitro*. Furthermore, concomitant administration of DNase 1, which dismantled NETs, or inhibition of peptidylarginine deiminase 4 (PAD4), an essential mediator of NET formation, suppressed the early inflammatory responses to Sev infection. Lastly, NETs primed bone marrow-derived cells to release cytokines that can amplify the inflammatory cascade.

## Introduction

Respiratory viral illnesses are common in early life. A majority of children with an initial episode of viral-induced bronchiolitis will have recurrent wheezing, prompting the association between viral infection, and subsequent development of asthma (1–3). In the mouse, infection with the paramyxovirus Sendai virus (Sev) causes a limited bronchiolitis followed by chronic persistent asthma traits characterized by mucous cell metaplasia and airway hyperreactivity (AHR) (4). We have previously shown that the asthma phenotype was attenuated in the absence of dipeptidyl peptidase I (DPPI), a cysteine protease with many immunomodulatory activities (5). This attenuation was accompanied by significant reduction in the number of alveolar neutrophils and local production of inflammatory cytokines in the acute phase of infection. Adoptive transfer of WT neutrophils into DPPI-deficient mice led to enhanced accumulation of DPPI-deficient neutrophils and inflammatory cytokine production in the alveolar space on day 4 post-infection (PI) and subsequent asthma phenotype development. The fact that DPPI-deficient neutrophils exhibited normal chemotaxis in response to various stimuli (5) but failed to accumulate at the site of inflammation (alveolar space) suggests a yet-to-be defined mechanism by which DPPI and DPPI-sufficient neutrophils modulate the early inflammatory responses to Sev.

Neutrophil extracellular traps (NETs) were initially described as a neutrophil defense mechanism to trap and kill bacteria (6). NETs have since been implicated in the pathogenesis of several inflammatory diseases, including bronchial asthma (7). Herein, we showed that Sev infection led to NET formation in the lung of WT mice. We also established that DPPI-deficient neutrophils exhibited a defect in NET formation *in vitro* in response to multiple stimuli. We hypothesized that the absence of DPPI attenuated NET formation in response to Sev infection, thus interrupting the inflammatory cascade and suppressing the ongoing leukocyte influx. Indeed, administration of DNase 1, which dismantled NETs, reduced free DNA–myeloperoxidase (MPO) complexes in the alveolar space and plasma, as well as attenuating the early inflammatory responses to Sev infection. Inhibition of peptidylarginine deiminase 4 (PAD4), an essential mediator of NET formation also suppressed alveolar leukocyte accumulation and cytokine production in the acute phase of infection, confirming the contribution of NETs to Sev-induced phenotype. Moreover, NETs from Sev-infected bronchoalveolar lavage fluid (BALF) stimulated bone marrow-derived cells (BMDCs) [dendritic cells (DCs) and macrophages] to release inflammatory cytokines.

## Materials and Methods

### Animals

Dipeptidyl peptidase I^−/−^ mice were generated in 129/SvJ as previously described (8) and backcrossed to C57BL/6J for >10 generations. Microsatellite genotyping showed that DPPI^−/−^ mice were 99.2% congenic with C57BL/6J mice (The Jackson Laboratory). WT C57BL/6J mice were obtained from The Jackson Laboratory. WT and DPPI^−/−^ mice were kept in pathogen-free environment until the time of Sev infection. All animal experiments were performed in compliance with federal laws and in strict accordance with the guidelines established by the Division of Comparative Medicine at Washington University in St. Louis.

### Viral Infection

Mice of 6–8 weeks of age were anesthetized with isoflurane and inoculated intranasally (i.n.) with 2,500 50% egg infectious dose of Sev (Fushimi strain) as previously described (5). Experimental infection with Sev was performed in biohazard containment facility. Some animals received DNase 1 (0.5 mg i.n./mouse) on days 0–2. Cl-amidine, a pan-PAD inhibitor (cat#506282, Calbiochem) was dissolved in DMSO/PBS (5% v/v) and administered i.p. at 10 mg/kg daily on days −2 and −1 prior to infection then twice a day on days 0–2 PI. Controls received the same volume of DMSO/PBS (5% v/v). At different time points, mice were sacrificed and their blood, BALF, and lung harvested for cell count, cytokine, MPO–DNA, and histologic analysis.

### Lung and BALF Analysis

After sacrifice, BALF was obtained as previously described (5). The cells were pelleted and analyzed by flow cytometry using: FITC anti-CD69 (cat# 561929; BD Pharmingen), PerCP anti-CD8a (cat# 45-0081-82; eBioscience), APC anti-CD4 (cat# 100516; BioLegend), PE anti-CD11c (cat#553802, BD Pharmingen), anti-Siglec-H (cat# MCA4647GA, AbD Serotec, Raleigh, NC, USA), APC anti-CD317 (cat# 127015, BioLegend), PerCP anti-CD11b (cat# 101229, BioLegend), FITC anti-rat IgG (cat# 712-095-150, Jackson ImmunoResearch Laboratories). In general, 10^6^ cells were blocked with the anti-FcR mAb 2.4G2, stained with the indicated antibodies for 20 min at 4°C and then washed and resuspended in FACS buffer for analysis. Flow cytometry was performed on a BD FACSCalibur™. Data analysis was performed using BD CellQuest™ Pro software. Cell-free BALF was subjected to cytokine analysis by cytometric bead arrays (CBA) or MPO–DNA complex analysis. The lung was snap frozen in OCT compound and examined for *in vivo* NET formation.

### *In Vivo* NET Detection

Cross sections (9 μm) of OCT-embedded frozen lung tissues were fixed in 4% paraformaldehyde, blocked in 8% BSA in PBS and incubated with the primary antibodies: anti-Histone H2B (1:100 dilution; Cat # SC-8651; Santa Cruz Biotechnology), anti-mouse MPO (1:100 dilution; Cat # HM1051BT; Hycult Biotech) followed by the appropriate rhodamine red- or FITC-conjugated secondary antibody (1:100–1:200; Jackson ImmunoResearch Laboratories). DNA was stained with DAPI. All images were acquired with QCapture software on a Nikon Eclipse microscope.

### *In Vitro* NET Induction

Neutrophils were isolated from bone marrow as previously described (9). Isolated neutrophils were seeded on Thermanox plastic coverslips (Cat # 174950, Thermo Fisher Scientific Inc.) or 5-mm round glass coverslips (Cat # 101413-528, VWR), placed in 24-well plates (75,000 cells/well) and incubated for 1 h at 37°C to allow adherence to coverslips. The following activating agents were used: LPS (10 μg/ml, Cat # L2762, Sigma-Aldrich), PMA (10 nM, cat# P8137, Sigma-Aldrich) or Sev (5,000), and rmTNF-α (10 μg/ml, cat# 410-MT, R&D Systems). After 30 min of stimulation, cells were fixed with 4% paraformaldehyde in PBS overnight and the DNA was stained with Sytox green (Cat # S7020, Invitrogen). NETs were visualized on a Nikon Eclipse fluorescence microscope and low magnification images (40×) were acquired with QCapture software on non-overlapping random images (7–11 separate fields/coverslips, derived from 3 wells/condition or genotype). NETs were manually identified on acquired images as Sytox-positive structures emanating from neutrophils with an overall length at least twice as long as the cell diameter (10) and expressed as percentage of neutrophils with released DNA. Each experiment was repeated at least three times.

### MPO–DNA ELISA

Anti-MPO antibody (5 μg/ml, cat# HM 1051BT, Hycult Biotech Inc) was used to coat 96-well plate overnight at 4°C. After three washes with washing buffer (PBS with 0.05% Tween 20), 20 μl of BALF or plasma samples were added to the wells with 80-μl incubation buffer containing a peroxidase-labeled anti-DNA monoclonal antibody (dilution 1:25, cat# 11544675001, Cell Death Detection ELISA, Roche). The plate was incubated for 2 h at room temperature. After three washes, 100 μl of peroxidase substrate (cat# DY999, R&D Systems) was added. The reaction was stopped with 1M H_2_SO_4_ and OD of samples measured at 450 nm (Molecular Devices SpectraMax Plus 384). Specific OD was obtained by subtracting total OD from background OD generated without the addition of peroxidase-labeled anti-DNA antibody (less than 10%).

### Cytokine Analysis

Cytokine concentrations in BALF samples and co-cultures were measured using the CBA for Mouse Inflammation Kit (cat# 552364, BD Biosciences), according to the manufacturer’s protocols.

### *In Vitro* Co-cultures

Murine bone marrow cells were cultured *in vitro* with GM-CSF (10 μg/ml, cat# PMC2015, Thermo Fisher Scientific) and Il-4 (5 μg/ml, cat# 404-ML/CF, R&D Systems) for 7 days. Cultured cells were plated in 24-well plates at 0.5 × 10^6^ cells/well. BALF collected on day 3 from Sev-infected mice were added at a final dilution of 44%. Supernatants were collected 2 days later and cytokine concentrations were analyzed by CBA. Baseline cytokine levels from BMDCs alone are subtracted from co-culture cytokine levels and values are presented as% of mean WT level, which is set at 100%.

### Statistical Analysis

Comparisons between two groups were performed by student’s *t*-test and comparisons between multiple groups (≥3) were performed by one-way ANOVA and Bonferroni’s correction for multiple comparisons was performed. Numerical data were expressed as mean ± SEM. *P* values <0.05 were considered significant.

## Results

### Sev Infection Induces NET Formation *In Vivo* and *In Vitro*

**Figure 1:**
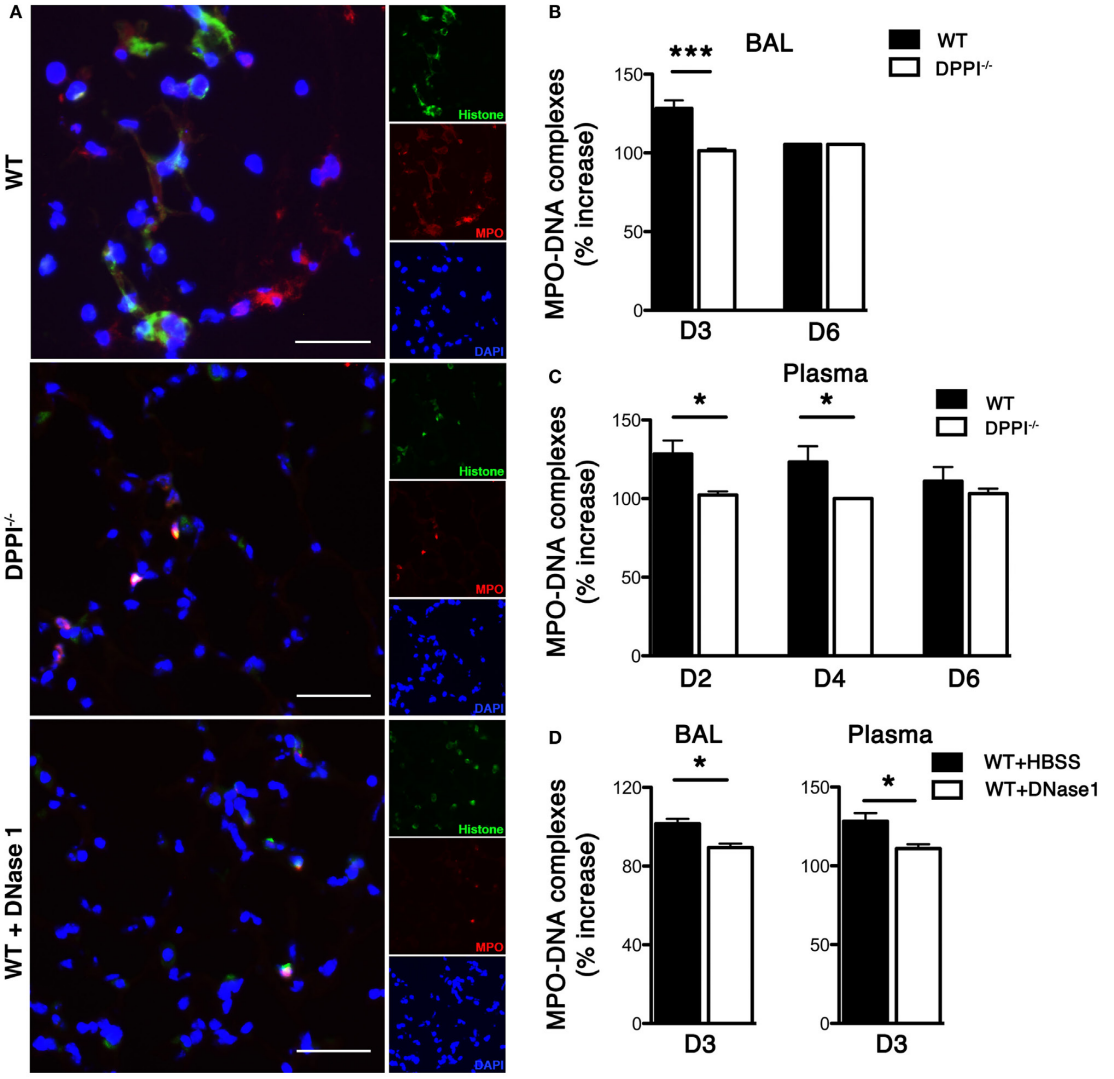
**Sev infection induces pulmonary NETs**. WT and DPPI^−/−^ mice were infected with Sev and their blood, lung, and BALF harvested on the indicated day PI. **(A)** Frozen lung sections from day 3 PI were stained for histone (green) and MPO (red). DAPI stained nuclei blue. The absence of DPPI and DNase 1 treatment suppressed NET formation. **(B–D)** BALF fluid and plasma from Sev-infected animals were collected on the indicated days and assayed for levels of MPO–DNA complexes. Values represent increase over baseline (obtained from uninfected mice) and expressed as mean ± SEM, *n* = at least five mice per genotype/treatment group. Scale bars = 100 μm. *P < 0.05, ***P < 0.001.

WT C57BL/6J and DPPI-deficient (DPPI^−/−^) mice were infected i.n. with 2,500 50% egg infectious dose of Sev as previously described (5). On day 3 PI, mice were sacrificed and their lung examined for NET formation by immunostaining with anti-histone and anti-MPO antibodies; DAPI stained DNA. NETs were easily detected in WT lung but greatly decreased in DPPI^−/−^ mice (Figure 1A). Concomitant administration of DNase 1, which dismantled NETs (11, 12), also suppressed Sev-induced NET formation in Sev-infected WT mice (Figure 1A).

To further quantify the NET content in Sev-infected mice we measured concentration of MPO–DNA complexes in the BALF and in plasma. The concentration of MPO–DNA complexes in BALF and plasma was increased in Sev-infected WT mice (Figures 1B,C). By contrast, we detected significantly lower levels of MPO–DNA complexes in the lung and plasma of Sev-infected DPPI^−/−^ mice on days 2–4 PI (Figures 1B,C). DNase 1 treatment also lowered the concentration of MPO–DNA complexes in BALF and plasma, confirming that the release of these complexes was partly NET dependent (Figure 1D).

**Figure 2:**
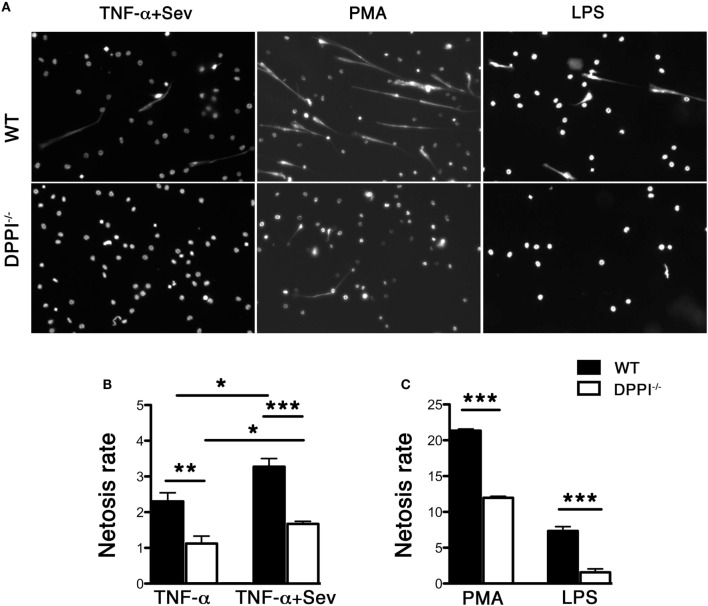
**Absence of DPPI attenuates NET formation *in vitro***. **(A)** Bone marrow-derived neutrophils from WT and DPPI^−/−^ mice were stimulated with TNF-α + Sev, PMA, or LPS for 30 min and stained for DNA with Sytox green. **(B,C)** Quantification of NETs with different stimuli (mean ± SEM derived from three separate experiments). *P < 0.05, **P < 0.01, ***P < 0.001.

*In vitro*, Sev enhanced NET release from TNF-primed, bone marrow-derived WT neutrophils (Figure 2). TNF-primed DPPI^−/−^ neutrophils generated lower levels of NETs in response to Sev (Figure 2) although the overall rate of Sev-induced NET formation was lower compared to PMA or LPS stimulation (Figure 2). Regardless of the stimulus, DPPI^−/−^ neutrophils consistently generated lower level of NETs, consistent with the *in vivo* results and previous studies (13, 14). Taken together these results suggest that Sev can directly induce NETs; however, in the lung milieu following Sev infection, several inflammatory cytokines likely contribute to or enhance Sev-induced NET formation.

### DNase 1 Attenuates the Early Sev-Induced Alveolar Inflammation

**Figure 3:**
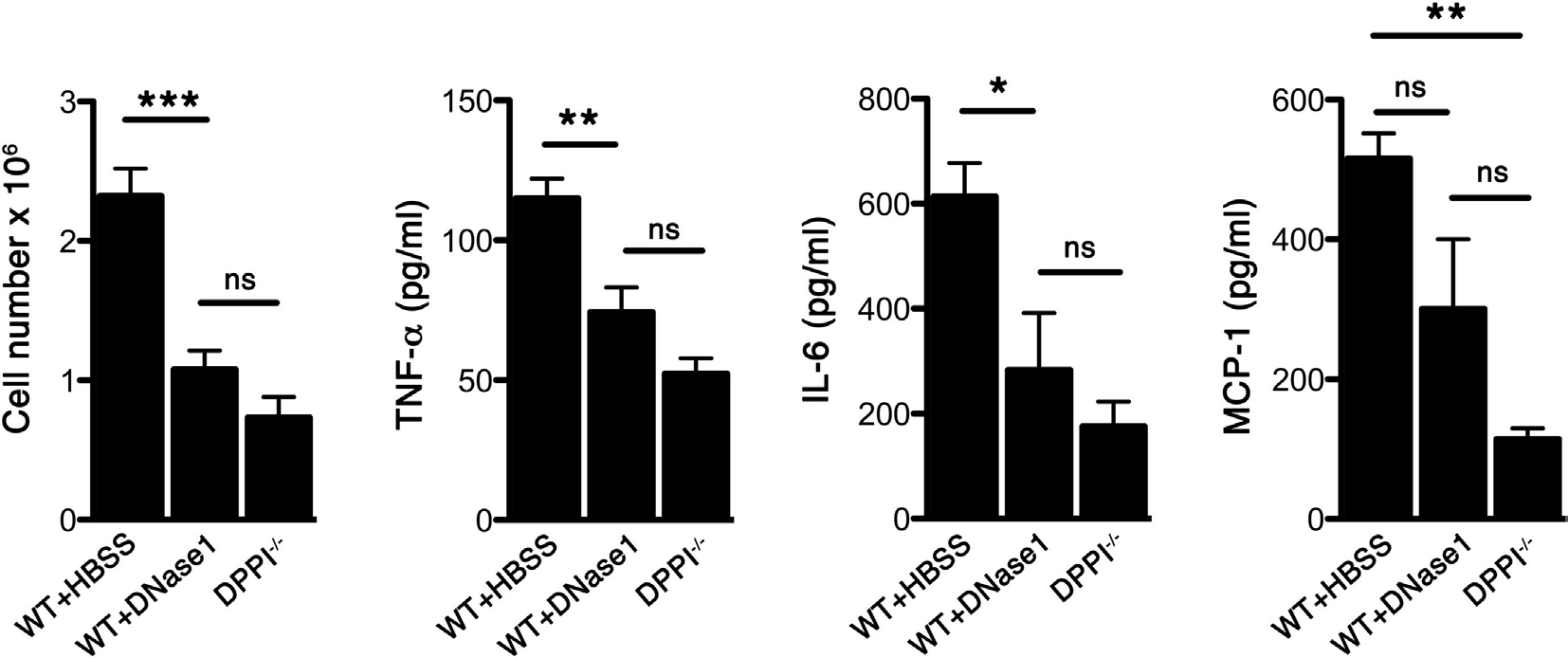
**DNase 1 treatment suppresses the acute Sev-induced inflammatory responses**. WT and DPPI^−/−^ mice were infected with Sev and their BALF harvested on day 3 PI. Some WT mice received DNase 1 i.n. on days 0–2 PI. The cells were enumerated and cell-free BALF assayed for cytokines by CBA. Values represent mean ± SEM, *n* = 4–5 mice per genotype/treatment group. *P < 0.05, **P < 0.01, ***P < 0.001, ns = not significant.

**Figure 4:**
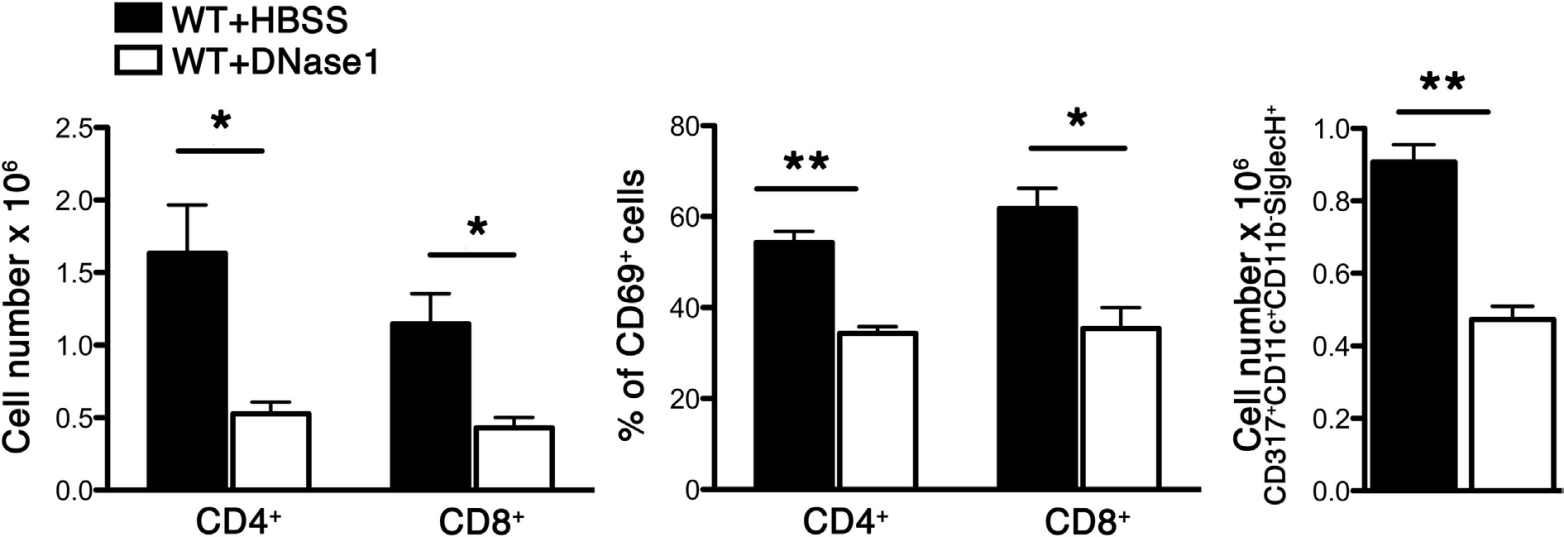
**DNase 1 treatment suppresses immune cell recruitment and activation**. WT mice were infected with Sev and treated i.n. with HBSS or DNase 1. Cells were harvested from BAL on day 3 PI and analyzed for CD4, CD8, CD69 (activation marker), and CD317+CD11c+CD11b-SiglecH+ (plasmacytoid dendritic cell markers) by multicolor FACS analysis. Values represent mean ± SEM, *n* = 4–5 mice/treatment group. *P < 0.05, **P < 0.01.

We have previously shown that Sev infection induced the expression of several cytokines/chemokines and absence of DPPI dampened this acute inflammatory response (5). To determine whether NETs modulated the acute response following Sev infection, we examined alveolar leukocyte accumulation and cytokine expression in WT mice treated with DNase 1. Administration of DNase 1 (i.n.) significantly reduced the total number of leukocytes recruited to the alveolar space (Figure 3). In addition, the levels of several BALF inflammatory cytokines, including TNF-α and IL-6 were suppressed with DNase 1 treatment (Figure 3). The extent of suppression with DNase1 treatment was equivalent to that observed with DPPI^−/−^ mice (Figure 3). DNase 1 treatment not only suppressed the acute cytokine response but also dampened the recruitment and activation of CD4^+^ and CD8^+^ T cells, as well as plasmacytoid DCs (CD317^+^ CD11c^+^ CD11b^−^ Siglec-H^+^) (Figure 4).

**Figure 5:**
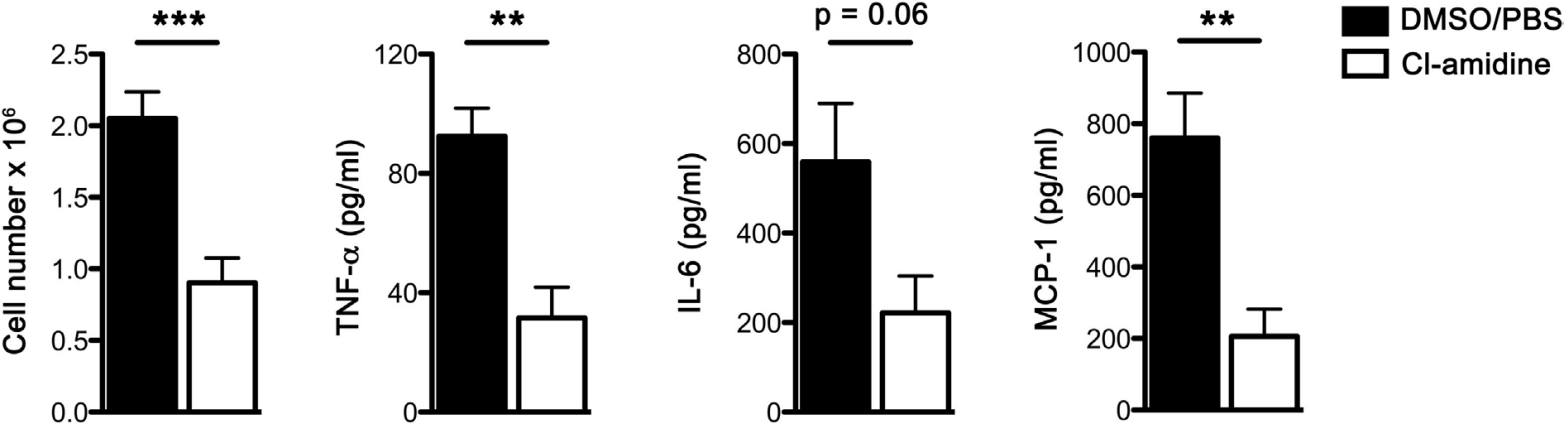
**Cl-amidine administration attenuates acute Sev-induced inflammatory responses**. WT mice were administered Cl-amidine i.p. daily 2 days prior to infection and treatment was continued (twice a day) until sacrifice as detailed in Section “Materials and Methods.” Controls received the same i.p. regimen of DMSO/PBS (5% v/v). On day 3 BALF was collected; cells were enumerated and cell-free BALF assayed for cytokines by CBA. Values represent mean ± SEM, *n* = 5 mice/treatment group. **P < 0.01; ***P = 0.001; note that IL-6 level in Cl-amidine treatment group approached statistical significance (*P* = 0.06).

To further confirm that NET plays a role in the early inflammatory responses to Sev infection, we administered Cl-amidine, a pan PAD inhibitor with preferential irreversible inactivation of the calcium bound form of PAD4 (15, 16) to Sev-infected mice. PAD4 catalyzes the conversion of specific arginine residues to citrulline (17) and is essential for the formation of NETs via PAD4-mediated citrullination of histones (18). Cl-amidine suppressed neutrophil accumulation and inflammatory cytokine release in the alveolar space (Figure 5). Combined with the DNase 1 treatment results, these findings further confirmed the involvement of NETs in early host responses to Sev.

### NET Containing BALF Stimulates Dendritic Cells/Macrophages to Release Inflammatory Cytokines

**Figure 6:**
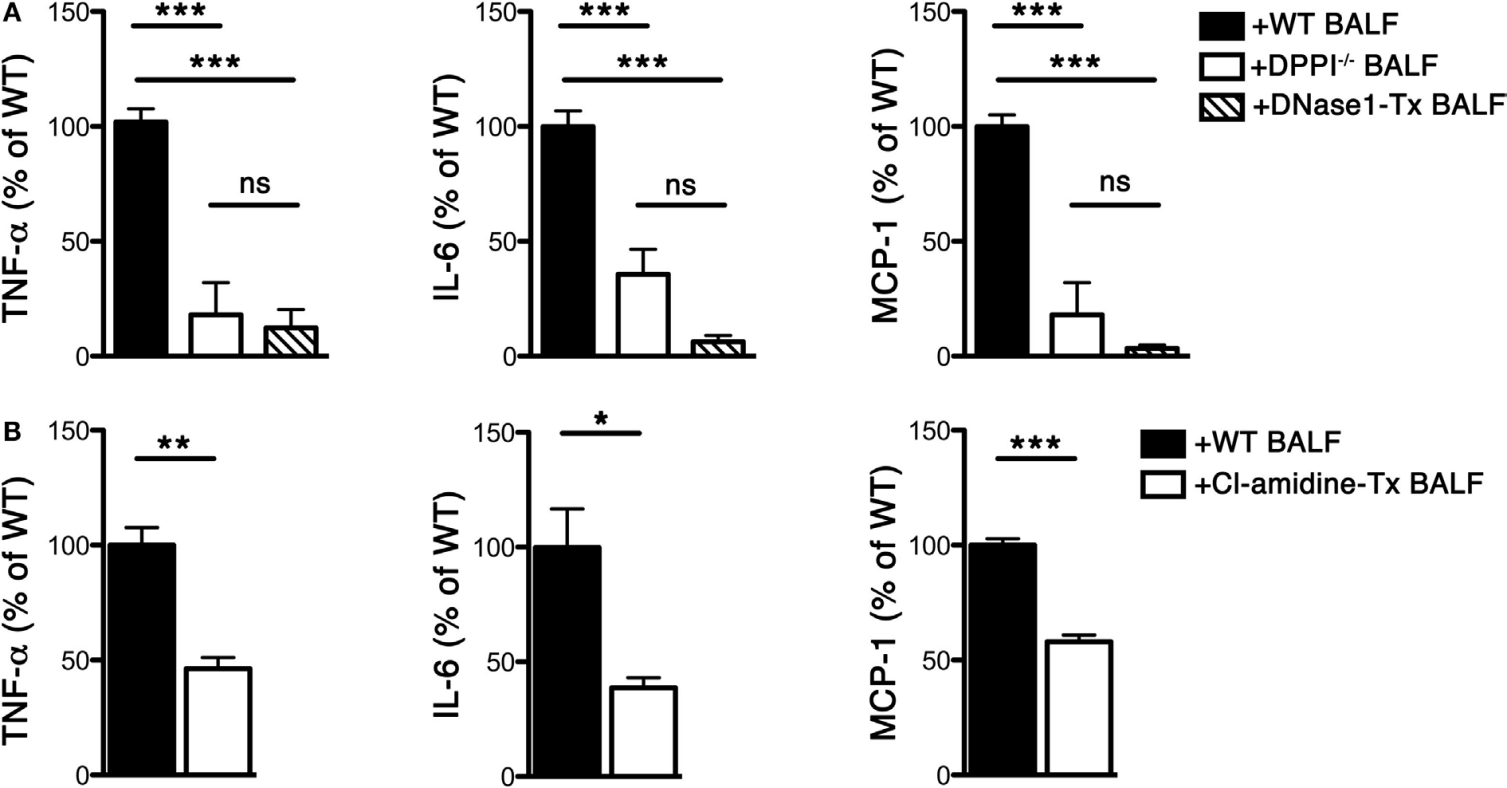
**BALF from Sev-infected mice stimulates cytokine release from BMDCs**. WT and DPPI^−/−^ mice were infected with Sev on day 0. Some WT mice were treated i.n. with DNase 1 or Cl-amidine as detailed in Section “Materials and Methods.” BALF was harvested on day 3 PI and cells were pelleted. The cell-free BALF from each genotype/treatment was added to BMDCs at a final dilution of 44%. The supernatant was collected after 48 h and assayed for cytokine levels with CBA. **(A)** Cytokine levels from co-cultures of BMDCs with BALF from WT, DPPI^−/−^, or DNase 1-treated (Tx) mice. **(B)** Cytokine levels from co-cultures of BMDCs with BALF from Cl-amidine-treated mice. Values represent mean ± SEM, *n* = 4–6 mice/treatment group. The mean of WT cytokine levels for each experimental condition was set at 100%; values are expressed as% of mean WT level. *P < 0.05, **P < 0.01, ***P < 0.001, ns = not significant.

Previous studies suggest that the early phase antiviral cytokine response correlates with chronic lung immunopathology, including the airway remodeling and AHR that accompany the asthma phenotype (19). In addition to Th2-types cytokines, Th1 cytokines, such as TNF-α, have also been shown to play an important role in the pathogenesis of asthma. TNF-α is increased in the sputum of patients with asthma (20, 21), aggrevates AHR (22) as well as recruit inflammatory cells in animals (23). Blockade of TNF-α significantly inhibits AHR and reduces airway inflammation (24). To this end, we examined whether BALF-derived NETs from Sev-infected mice modulated TNF-α (and other inflammatory cytokine) release from *in vitro* GM-CSF-induced BMDCs that comprise conventional DCs and macrophages (25). We observed that NET-containing BALF from WT Sev-infected mice directly stimulated BMDCs to release substantial amount of TNF-α (as well as IL-6 and MCP-1) while BALF from DPPI^−/−^ mice and animals treated with DNase 1 was less efficient (Figure 6A). Cl-amidine treatment also significantly attenuated the release of inflammatory cytokines by BALF-stimulated BMDCs (Figure 6B). Taken together, these above findings suggest that NETs released by recruited neutrophils play an important role in shaping the early immune response that likely impacts the Sev-induced chronic lung immunopathology.

## Discussion

Neutrophil extracellular traps can have anti- and pro-inflammatory effects on disease pathogenesis. On the one hand, NETs have been shown to promote the resolution of neutrophilic inflammation in monosodium urate crystal-induced arthritis (gout) (26). On the other hand, NETs are increasingly implicated in driving the pathogenesis of various diseases (11, 27–31). Their role in asthma development, however, remains largely unknown. Asthma is considered an inflammatory disease involving different mediators and cell types, with eosinophil predominance. Non-eosinophilic asthma, however, comprises 50% of asthma and is often associated with accumulation of neutrophils in the airways, possibly due to bacterial endotoxin exposure as well as viral infections (32). Neutrophils are often present in acute asthma exacerbations (33) and noted in specific circumstances, such as status asthmaticus (34) but their role in asthma pathogenesis has not been extensively explored. A more recent study suggests that NETs are generated in human atopic asthmatic airways (35); however, their pathophysiologic role in allergic asthma also remains undefined. Using a physiologic murine model of Sev-induced asthma phenotype, we previously established a critical role for neutrophils and neutrophil-associated DPPI in the disease pathogenesis (5). In the studies herein, we further extend the investigation to show that neutrophil-derived DPPI is an important mediator of NET formation and NETs mediate the early inflammatory responses in Sev infection.

In addition to Sev-induced asthma phenotype, the absence of DPPI protects against various inflammatory conditions, including preclinical models of rheumatoid arthritis (36), sepsis (37), abdominal aortic aneurysm (38), and ANCA-associated glomerulonephritis (39). DPPI deficiency also attenuates early atherosclerotic lesion in LDL-receptor-deficient mice (40). A recent report established that the absence of DPPI abrogates *in vitro* NET formation (13). DPPI is a ubiquitously expressed cysteine protease that is required for the expression of neutrophil serine proteases (NSPs), neutrophil elastase (NE), cathepsin G (CG) and proteinase 3 (PR3) in mature neutrophils (13, 36, 41). The role of NSPs in NET formation is well accepted (42). It is suggested that NE, but not CG or PR3, translocates to the nucleus where it contributes to chromatin decondensation by cleaving histones, an essential step in NET formation (43, 44). However, Martinod et al. recently showed that NE is dispensable for *in vitro* and *in vivo* NETs (45). Consistent with these results, we found that murine NE-deficient neutrophils release normal levels of NETs *in vitro* in response to various stimuli while the absence of NE and PR3 results in a NET defect similar to that observed with DPPI-deficient neutrophils (14). These discrepancies may reflect species-dependent requirements for NET formation, as previous studies used human neutrophils and NSP inhibitors.

Airway remodeling is an important histologic and structural change seen in asthma. It is a complex and incompletely understood process, involving several cell types and mediators. Neutrophils have been linked to airway remodeling presumably through their ability to release proteases, including metalloproteases and NE that induce degradation of extracellular matrix and modulate inflammatory cell trafficking (46–48). Herein, we show that DNase 1 treatment significantly reduced the recruitment and activation of leukocytes, including CD4^+^ and CD8^+^ T cells, in the acute phase PI and suppressed inflammatory cytokine release. BALF from DNase 1- and Cl-amidine-treated animals was also less efficient at stimulating bone marrow-derived macrophages and DCs from releasing inflammatory cytokines. Taken together these results suggest that NETs released during the acute phase of Sev infection induce airway inflammation, leading to the elaboration of inflammatory cytokines that further recruit and activate immune cells, thus promoting the eventual chronic asthma phenotype.

In summary, these findings suggest that controlling neutrophil activation and NET formation in non-eosinophilic asthma may attenuate acute exacerbations and potentially prevent further airway remodeling.

## Author Contributions

AA and LS designed and performed the experiments, and analyzed the data; AA, LS, and CP wrote the manuscript. All authors approved the final version of the manuscript.

## Conflict of Interest Statement

The authors declare that the research was conducted in the absence of any commercial or financial relationships that could be construed as a potential conflict of interest.
